# Guidelines for care in sexual violence: the role of medical training: Number 4 -April 2021

**DOI:** 10.1055/s-0041-1729994

**Published:** 2021-05-12

**Authors:** Anibal Eusébio Faundes Latham, Cristião Fernando Rosas, Helena Borges Martins da Silva Paro, Michele Lopes Pedrosa, Rivaldo Mendes de Albuquerque, Robinson Dias de Medeiros

**Affiliations:** 1 Universidade Estadual de Campinas, Campinas, SP, Brazil; 2 Maternidade Escola Vila Nova Cachoeirinha, São Paulo, SP, Brazil; 3 Universidade Federal de Uberlândia, Uberlândia, MG, Brazil; 4 Universidade Federal do Rio de Janeiro, Rio de Janeiro, RJ, Brazil; 5 Universidade de Pernambuco, Recife, PE, Brazil; 6 Universidade Federal do Rio Grande do Norte, Natal, RN, Brazil

## Keypoints

- Despite the high frequency and morbidity and mortality associated with violence against women, this problem is often overlooked in both medical and tocogynecological practice.
- Violence against women is highly prevalent. Globally, one in every three women in the world has experienced physical or sexual violence from an intimate partner or any other abuser in their lifetime. It is a hidden violence that needs to be identified in a way that allows victims' embracement and provision of appropriate care.
- In Brazil, a rape occurs every eight minutes. Most victims of sexual violence result not only in emergency health problems, but also have negative and lasting consequences to health in general and especially to sexual and reproductive health. These repercussions are also observed in women who suffer other types of violence, such as physical and psychological.
- User embracement and quality and humanized care have a positive impact on the reduction of health problems resulting from sexual violence, as well as on the guarantee of human, sexual and reproductive rights.
- Being at people's side in situations of sexual violence in order to guarantee their basic rights to health and dignity is a professional duty. These rights may include termination of rape-related pregnancy.
- In general, the discussion of human, sexual and reproductive rights is not included in the curricular structures of health professionals' training. Topics such as gender violence, attention to health problems resulting from sexual violence and abortion provided for by law are not addressed in undergraduate medicine courses or, when they are, it is in superficial manner.
- Medical courses, medical residency and Master's and PhD postgraduate programs do not include health care in situations of sexual violence and abortion provided for by law.
- Due to professional difficulty or lack of knowledge, conscientious objection is often invoked when handling cases of victims of sexual violence. The allegation of conscientious objection can create barriers to care, which contributes to higher risk and vulnerability of girls and women in situations of sexual violence in Brazil.

## Recommendations

- For the guarantee of appropriate care to girls and women in situations of sexual violence, issues related to sexual and reproductive health must be included in the curricula of undergraduate courses in Medicine and other health areas, Postgraduate courses, Medical Residency Programs in Gynecology and Obstetrics, and in continuing education activities.
- Medical courses and Medical Residency Programs in Gynecology and Obstetrics must provide opportunities for students' theoretical and practical learning, so they can develop professional competences related to comprehensive care for people in situations of sexual violence and abortion provided for by law.
- **The following are essential competences of the doctor** that must be developed during the undergraduate Medical course: assistance to girls and women in situations of sexual violence with knowledge of the ethical and legal aspects related to sexual violence and abortion provided for by law (crimes against sexual dignity, professional secrecy and confidentiality, limits of conscientious objection).
- Recognition of signs of violence against women and the adoption of preventive measures and appropriate treatment.
- Embracement, counseling and prescription of prophylaxis against sexually transmitted infections, counseling and prescription of emergency hormonal contraception to girls and women in situations of sexual violence.
- Appropriate referral to the health network, social services and public safety of girls and women in situations of sexual violence and abortion provided for by law, when applicable.
- **The following are essential competences of the gynecologist and obstetrician** that must be developed over the three years of specialization: user embracement, counseling, adequate record of history and physical examination in medical records, prescription of prophylaxis against sexually transmitted infections, counseling and prescription of hormonal and non-hormonal (intrauterine device) emergency contraception to girls and women in situations of sexual violence and collection of forensic evidence of sexual violence, when relevant.
- Advice on risks, benefits, complications and indications of medical abortion and surgical abortion in the different stages of pregnancy.
- Interdisciplinary management of complications related to abortion provided for by law (hemorrhage, infection, sepsis, shock).
- Counseling and obtaining consent to induce fetal death and abortion after 22 weeks of pregnancy in cases provided for by law.

## Clinical context

### Sexual and reproductive rights

Women's human rights include their rights to have control and decide freely and responsibly on issues related to sexuality and sexual and reproductive health, free from coercion, discrimination and violence. 1 These rights started to be included as a priority agenda from the end of the 20 ^th^ century in several international conferences. The 2 ^nd^ World Conference on Human Rights held in Vienna in 1993 made history by stating that the rights of women and girls are inalienable and constitute an integral and indivisible part of universal human rights. During the Conference, abuses in the private sphere - rape and domestic violence - were defined as crimes against the dignity of the human person. The final document also called on states to ratify the Convention on the Elimination of Discrimination against Women, approved by the General Assembly of the United Nations (UN) in 1979, which aimed to reduce and eradicate violations in the field of sexuality and reproduction. 2

In the year following the Vienna Conference, the International Conference on Population and Development (ICPD) that took place in Cairo was a historic landmark for sexual and reproductive rights. At that time, the previously hegemonic discourse of limiting population growth as a means of reducing poverty and social inequalities was replaced by another paradigm: that of sexual and reproductive rights. In opposition to control policies, the Cairo Platform recognized “the basic right of all couples and individuals to decide freely and responsibly on the number of children, birth spacing and the interval between them and to have information and means to do so”, including “the right to make decisions regarding reproduction without suffering discrimination, coercion or violence”. 3

In 1995, at the IV Conference on Women held in Beijing, progress was made in the understanding that autonomy, equality and sexual and reproductive security are essential in guaranteeing the human rights of girls and women, who need to “have control over aspects relating to sexuality, including their sexual and reproductive health, and to decide freely on these issues, without being subject to coercion, discrimination or violence”. The Conference document further recommends that countries review laws that impose punishments on women who perform illegal abortions, in order to emphasize the serious public health problem associated with clandestine abortions. 4

The Brazilian State actively participated in the construction of these and other international documents and treaties addressing the guarantee of human, sexual and reproductive rights of girls and women. As a signatory to these documents, Brazil must make efforts so the deliberations defined in these meetings influence the internal legal system of the country. Several recommendations present in these documents are materialized in the Federal Constitution of 1988 and other infra-constitutional laws. 5

### Violence against women

The UN defines gender violence as “any act of gender-based violence that results or could result in physical, sexual or psychological harm or suffering to a woman, including the threat of such acts, coercion, arbitrary deprivation of liberty, whether in the public or private sphere”. 6 Violence against women, a serious violation of human rights, is an important public health problem, as it is a high prevalence determinant of illnesses and deaths worldwide.

According to the World Health Organization (WHO), 7 35.6% of women suffer physical or sexual violence throughout their lives, with a variation of 32.7% between the set of developed countries and 45.6% in the African continent. The probability of physical or sexual aggression by an intimate partner is 30.0%, more than four times higher than the probability of physical or sexual aggression by an unknown aggressor, which is approximately 7%. 7

In Brazil, 3,730 women were murdered (one every two hours) in 2019. 8 Due to the higher victimization of black women, between 2008 and 2018 there was a 4.3% increase in the female homicide rate: while the homicide rate of non-black women fell by 11.7%, the rate among black women increased by 12.4%. 9

In relation to sexual violence, Brazil has one of the highest prevalence rates of rape in the world, with one case occurring every eight minutes, according to data from police records. In addition, the estimated under-reporting is close to 90% of cases, 10 which means that these numbers can be up to ten times higher. 9 10

Violence against women is associated with physical and mental damage with short- and long-term manifestations. Studies that evaluated the chance of illness among women victims of violence show a 1.52 times higher chance of HIV infection, 2.16 times of induced abortion, 1.56 times of occurrence of depressive symptoms and 4.54 times of suicide. 7 Women who have an intimate partner are three times more likely to suffer physical injury than those who do not. 7

Despite such alarming numbers, health system professionals are unprepared to identify girls and women in situations of violence and provide user embracement. Health services, whether emergencies, primary care units or specialized outpatient clinics, receive a large number of women in situation of violence or who have experienced gender violence in the course of their lives. 11 12 A systematic review conducted with studies in different health care settings identified that 40–70% of women attended reported violent experiences throughout their lives. 11 Likewise, Schraiber et al., 12 in a study conducted in primary care units in Greater São Paulo, observed that 70% of women had already suffered one or more episodes of violence.

In this sense, health professionals are in a strategic position to detect situations of violence against women. 13 14 However, despite its high frequency and serious implications for women's health, professionals in the health system are not sensitive to the issue of sexual violence. In the study by Schraiber et al., 12 only 7% of medical records indicated the violence experienced. This fact is possibly related to the lack of theoretical and practical tools during professional training.

The systematic exclusion of topics related to women's health and sexual and reproductive rights has been observed in Brazilian medical training over the years. 15 16 General practitioners, gynecologists and obstetricians notably need to be better informed about this issue. If professionals do not know how to identify the symptoms and signs that make them suspect that a woman suffers this type of violence, they will be unable to identify the problem and provide the necessary care and embracement.

The lack of contact with the care of girls and women who need abortion provided for by law during their professional training can also lead to the scarcity of an already limited number of Brazilian services, 17 since ignorance contributes to allegations of conscientious objection among doctors. The theoretical and practical inclusion of sexual violence against women can certainly contribute to reduce the consequences of this violence on the physical and mental health and quality of life of Brazilian girls and women.

### Abortion provided for by law

In Brazil, abortion is allowed in cases of risk of death for women, when pregnancy results from rape 18 and in cases of fetal anencephaly. 19 Although access to termination of pregnancy is women's right, in practice, when they seek health services to perform abortion, access is not always easy. Madeiro and Diniz 17 observed that in Brazil, between 2013 and 2015, only 37 out of the 68 services performed interruption of pregnancy resulting from rape and these were concentrated in the Southeast and Northeast regions of the country. Most of these services (80%) had been implemented until year 2005. After that period, few services were implemented and some stopped working. Seven states did not have an active service available at the time of the study. 17

Added to the difficulty of access is the lack of doctors prepared to interrupt the pregnancy, especially in cases resulting from rape. This happens both because of unfamiliarity with the protocols and techniques of this procedure and the lack of knowledge of the associated ethical and legal issues. In a study of 1,174 medical students 20 , 50.8% of them reported having conscientious objection to interrupt pregnancy resulting from rape, and this proportion was much lower for cases of fetal anencephaly (31.6%) and risk of maternal death (13.2%). Among students who refused to perform abortion, most (72.5%) would not refer these women to another professional and would not explain treatment options either. 20

A survey of 404 residents of gynecology and obstetrics at 21 teaching hospitals found that they had little knowledge of medical abortion. However, being in more advanced years of residence and having participated in the care of women diagnosed with induced or probably induced abortion was associated with a greater knowledge of these professionals on the issue. 21

In 2013, Cacique et al. 22 systematized all scientific publications containing the theme of abortion performed in Brazil between 2001 and 2011. In the studies analyzed, although most gynecologists and obstetricians had knowledge about the legal permission for abortion, knowledge about the necessary documentation, the breach of confidentiality associated with denunciation of the woman who practiced illegal abortion, and records of suspected illegal abortion in the medical record was limited. In addition, a significant portion of specialists had never been trained to induce abortion. 22

## What is the importance of including learning opportunities and care services for women victims of violence and legal abortion in university hospitals in Brazil?

Brazil is a signatory to several international documents that aim to guarantee the rights and sexual and reproductive health of girls and women. To this end, the offer of health actions focused on issues associated with this field of women's health is essential, including promotion, prevention and care activities.

Comprehensive and humanized care for women's sexual and reproductive health will only be implemented in public and private, outpatient and hospital daily services if there is massive investment in health professional training, so they can master the care protocols, ethical and social aspects necessary to the guarantee of constitutional rights. Discussions on topics related to sexual and reproductive health in undergraduate, postgraduate and continuing education courses are essential, with emphasis on biomedical, bioethical and legal issues associated with violence against women, including victims of sexual violence, and for the embracement and proper management of cases of abortion provided by law, as well as care for women who practice illegal abortion. The same way that it is the duty of the State to provide health services for the care of women victims of violence and women entitled to termination of pregnancy resulting from rape, risk of maternal death and anencephaly, it is the duty of educational institutions to prepare their students according to determinations of the Brazilian legislation and national and international norms and protocols.

In view of the above, the Brazilian Federation of Associations of Gynecology and Obstetrics (Febrasgo) recommends that teachers of gynecology and obstetrics in medical courses in Brazil, coordinators of Medical Residency and Postgraduate Programs in Gynecology and Obstetrics, as well as Commissions and Scientific Boards of Associated Societies comply with the ethical and constitutional obligation to offer learning opportunities and care services to women victims of violence and legal abortion in the corresponding university hospitals, thereby avoiding complications associated with these injuries, with emphasis on the search for unsafe abortion. This measure has a fundamental role in the training of human resources in health and seeks to guarantee the multiplication of good practices in order that sexual and reproductive health demands are met by medical professionals from the various public and private services in the country.

## What are the competences related to the care of girls and women in situations of sexual violence that should be developed by medical courses students?

In order to ensure consistency and coherence with the sexual and reproductive health needs of girls and women in situations of sexual violence in the curricula of medical courses in Brazil, the National Specialized Commission on Sexual Violence and Abortion foreseen in the Febrasgo Law recommends special attention from educators and managers to the competences required of general practitioners.

## Competences of medical course graduates

Medical courses should offer theoretical and practical learning opportunities for students in order to enable them to:

- Demonstrate knowledge about ethical and legal care-related aspects of girls and women in situations of sexual violence and abortion provided for by law (crimes against sexual dignity, professional secrecy and confidentiality, limits of conscientious objection);
- Recognize the signs of violence against women and adopt appropriate preventive and treatment measures;
- Provide user embracement, perform adequate record of anamnesis and physical examination in medical records, counseling and prescription of prophylaxis against sexually transmitted infections, counseling and prescription of emergency hormonal contraception to girls and women in situations of sexual violence;
- Demonstrate knowledge and provide counseling to girls and women regarding alternatives to pregnancy resulting from rape;
- Make appropriate referrals of girls and women in situations of sexual violence to the health network, social services and public safety and abortion provided for by law, when applicable.

## Which competences related to the care of girls and women in situations of sexual violence should be developed by gynecology and obstetrics first-year residents?

In order to ensure consistency and coherence of Gynecology and Obstetrics Medical Residency Programs with the sexual and reproductive health needs of girls and women in situations of sexual violence in Brazil, the National Specialized Commission on Sexual Violence and Abortion foreseen in the Febrasgo Law recommends special attention from educators and managers to the competences required of gynecology and obstetrics specialists.

Gynecology and Obstetrics Residency Programs should provide first-year residents with theoretical and practical learning opportunities in order to enable them to:

- Provide user embracement, perform adequate record of anamnesis and general and gynecological physical examination in medical records, counseling and prescription of prophylaxis against sexually transmitted infections, counseling and prescription of hormonal and non-hormonal (intrauterine device) emergency contraception for girls and women in situations of sexual violence, collection and preservation of forensic evidence, when pertinent;
- Offer counseling on risks, benefits, complications and indications of medical abortion and first-trimester surgical abortion;
- Obtain the informed consent of girls and women for the first trimester abortion provided for by law.

## Which competences related to the care of girls and women in situations of sexual violence should be developed by gynecology and obstetrics second-year residents?

Gynecology and Obstetrics Residency Programs should provide second-year residents with theoretical and practical learning opportunities in order to enable them to:

- Prescribe and properly guide the use of misoprostol for medical abortion;
- Assist girls and women hospitalized for medical induction of abortion provided for by law in the second trimester of pregnancy;
- Perform manual intrauterine aspiration in legal abortions in pregnancies of up to 14 weeks.

## Which competences related to the care of girls and women in situations of sexual violence should be developed by gynecology and obstetrics third-year residents?

Gynecology and Obstetrics Residency Programs should offer theoretical and practical learning opportunities to third-year residents in order to enable them to:

- Perform interdisciplinary management of complications related to abortion (hemorrhage, infection, sepsis and shock);
- Perform surgical management of abortion provided for by law after 14 weeks of pregnancy (dilation and evacuation);
- Demonstrate knowledge about the technical, ethical and legal aspects related to the induction of fetal death in cases of abortion provided for by law;
- Offer counseling and obtain consent to induce fetal death and abortion after 22 weeks of pregnancy in cases provided for by law.

## Final considerations

Health professionals need to be prepared for the embracement and comprehensive care of women, including all demands related to sexual and reproductive health and demands for abortion provided for by law. There is no quality health care for women without adequately prepared health professionals committed to sexual and reproductive issues, with emphasis on care for victims of sexual violence and abortion in cases provided for by law. The National Specialized Commission on Sexual Violence and Abortion provided for by law strongly recommends the inclusion of discussions on sexual and reproductive health and rights in undergraduate courses, continuing education activities, Medical Residency Programs and Postgraduate Programs in Gynecology and Obstetrics. Attention to the essential competences listed in this position is essential and urgent in order to achieve a situation of care to women's health in accordance with good practices defined by national and international regulations.

National Specialty Commission for Sexual Violence and Abortion Provided for by Law of the Brazilian Federation of Gynecology and Obstetrics Associations (FEBRASGO)

President:

Robinson Dias de Medeiros

Vice-President:

Cristião Fernando Rosas

Secretary:

Helena Borges Martins da Silva Paro

Members:

Aline Veras Morais Brilhante

Anibal Eusébio Faundes Latham

Débora Fernandes Britto

Edison Luiz Almeida Tizzot

Isabelle Cantídio Fernandes Diógenes

Kenia Zimmerer Vieira

Michele Lopes Pedrosa

Osmar Ribeiro Colás

Rivaldo Mendes de Albuquerque

Rosires Pereira de Andrade

Suely de Souza Resende

Zélia Maria Campos
